# Characteristic mango price forecasting using combined deep-learning optimization model

**DOI:** 10.1371/journal.pone.0283584

**Published:** 2023-04-13

**Authors:** Xiaoya Ma, Jin Tong, Wu Huang, Haitao Lin

**Affiliations:** 1 Department of Logistics Management and Engineering, Nanning Normal University, Nanning, Guangxi, China; 2 Guangxi Key Lab of Human–Machine Interaction and Intelligent Decision, Nanning Normal University, Nanning, Guangxi, China; 3 School of Business Administration, Zhongnan University of Economics and Law, Wuhan, Hubei, China; 4 Yuxi Normal University, Yuxi, Yunnan, China; Jeonbuk National University, REPUBLIC OF KOREA

## Abstract

Accurate product price forecasting is helpful for scientific decision-making and precise industrial planning. As a characteristic fruit that drives regional development, mango price prediction is of great significance to several economies. However, owing to the strong volatility of mango prices, forecasting is vulnerable to uncertainties and is very challenging. In this study, a deep-learning combination forecasting model based on a back-propagation (BP) long short-term memory (LSTM) neural network is proposed. Using daily mango price data from a large fruit wholesale trading center in China from January 2^nd^, 2014, to April 18^th^, 2022, mango price changes are learned and predicted to support the fruit industry. The results show that the root mean-square error, mean absolute percentage error, and the R^2^ determination coefficient of the BP-LSTM combination model are 0.0175, 0.14%, and 0.9998, respectively. The prediction results of the combined model are better than those of the separate BP and LSTM models. Furthermore, it best fits the actual price profile and has better generalizability.

## 1 Introduction

With the desire to improve living standards worldwide, the demand for fruit consumption continues to grow. Economically, fruits have a high price elasticity of demand, causing prices to fluctuate wildly and resulting in market instability. During the COVID-19 pandemic in particular, drastic fruit price fluctuations were frequent, and residential consumption was disrupted, which posed great challenges to fruit farmers and related enterprises. A consequential objective of understanding fruit price fluctuation rules is to alleviate the impact of imbalanced supplies and demand on the fruit market to some extent.

The mango is a common tropical and subtropical fruit and is the focus of this report. According to China’s Bureau of Statistics, in 2021 the world’s leading mango-producing regions included India, southern China, and Southeast Asian countries. Among these, India’s annual output continues to be about 16.34M tons, accounting for about 42.2% of the world’s total output. Southern China’s 4.35M tons account for 11.2%, Thailand’s 2.60M tons account for 6.5%, and The Philippines’ amount accounts for 3.6%. As such, in these regions, mango production is a pillar industry with local characteristics, and these and other developing countries rely on developing industries. However, owing to many external factors, prices continue to fluctuate, resulting in adverse effects to mango producers, market operators, and consumers. Forecasting is the basis of decision-making, and making accurate price forecasts is crucial to industrial planning, development, and operations.

Traditional methods either predict prices with strong subjectivity or excessively rely on linear pricing relationships, which results in low prediction accuracy [1]. Compared with traditional prediction methods, neural network methods in deep learning have proven to achieve better data prediction. Neural networks, with powerful nonlinear mapping capabilities, can fit data relationships more accurately. Our research uses daily mango prices of large fruit wholesale trading centers from January 2nd, 2014, to April 18th, 2022 as empirical data. According to the role of deep learning in forecasting problems, important features were extracted from high-dimensional data and the selected features were input into the neural network prediction model, and the BP-LSTM (back-propagation (BP) and long short-term memory (LSTM) neural-network combined model) neural network price forecasting combination model is proposed to forecast mango price. The purpose is to research the application of neural network prediction methods in the field of deep learning in the price prediction of characteristic agricultural products.

Based on BP neural network and LSTM neural network prediction methods, this paper proposes a combined optimization BP-LSMT neural network price prediction model for mango, a special fruit, which enriches the research methods in the field of characteristic agricultural products price prediction. This study fills a research gap pertaining to the lack of reliable mango price-prediction capability in existing agricultural models. Furthermore, the model is highly representative of the tropical and subtropical Asian regions.

## 2 Related literature

### 2.1 Literature overview

Regional fruit price forecasting is commonplace, and it is crucial to business decision-making in those areas. According to the characteristics of various agricultural products, the type of forecasting can be either long-term [2] or short-term [3–6]. However, for fruits, it is mostly short-term, based on seasonality, periodicity, and perishability. The key quantitative analytical methods include econometric, statistical, intelligent analysis, and combined methods [7]. For example, the autoregressive integrated moving average (ARIMA) model, which extends the autoregressive moving average (ARMA) model, is widely used for agricultural econometric time-series forecasting. Tatarintsev et al. [8] established an ARIMA-based price-change prediction model for sugar crops, and Shyian et al. [9] did so for short-term milk price prediction. Hence, it is known to work. For fruit crops, Niu Guicao et al. [10] used an ARIMA model to make short-term price predictions of the Hebei pear.

Machine-learning capabilities now offer a variety of new and powerful forecasting techniques, particularly unsupervised deep-learning types. Convolutional Neural Networks (CNNs), Recurrent Neural Networks (RNNs), and Deep Belief Networks are exemplary methods. Tasks of prediction, classification, and pattern recognition are routinely accomplished in many different fields with deep learning, and they have resulted in many benefits to science and society. This is particularly true for stock-price forecasting [11–13]. For example, Dong et al. [14] examined the stock-price predictability of the Hausdorff fractional gray model, which was built upon a CNN. Yu et al. [15] did the same using a locally linear embedding BP model. Hence, a deep-learning application for mango-specific agricultural product price prediction is overdue.

The BP neural network (BPNN) is a classic model that has the characteristics of self-adaptation, -organization, and -learning. Li et al. [16] used a gray-type BPNN to predict the grain crop yield in Henan Province. Notably, BPNNs have unique advantages with their embedded stochastic gradient descent algorithm, which arrives at accurate predictions in the face of nearly random patterns. Long-term price predictions are also within the realm of the BPNN, particularly when applying weights to the multi-dimensional factors affecting price over time [17]. In addition to price forecasting tasks [18, 19], BPNNs are often combined with other methods [20, 21] to obtain even higher accuracy with other complicated problems.

The LSTM, first proposed by Hochreiter and Schmidhuber (1997), is a special RNN that comprehensively learns time-series information to make short- and long-term predictions. Ye et al. [22] constructed one for stock price forecasting based on an autoregressive synthetic moving average and combined it with a BPNN. LSTM models based on mean absolute error and mean-square error criteria tend to show high accuracy with time-series predictions. In terms of agricultural price forecasting, Fang Xueqing et al. [7] made short-term price forecasts for Fuji apples using the ensemble empirical mode decomposition LSTM, and Wang Xiaolei et al. [23] made apple price predictions based on an LSTM that used a generalized autoregressive conditional heteroskedasticity method. In terms of agricultural product price forecasting, it is important to note that the selection focus of forecasting methods has gradually shifted from traditional regression methods [24, 25] to deep-learning models [26]. All of these examples support the potential of using deep learning to support the mango price prediction needs of our developing economies.

### 2.2 Research review

In this research, the Web-of-Science and Elsevier’s EI databases were searched using “and” connectors for key terms, such as “agricultural products,” “price forecasting,” and “agricultural price forecast,” and a total of 31 well-fitted journal articles were found. Unfortunately, most agricultural price forecasting publications focus on meat, vegetables, grains, etc. [27–29]. Therefore, we looked for model types and functional characteristics that could be extended to mango price prediction.

## 3 Research method

### 3.1 BPNN

**Fig 1:**
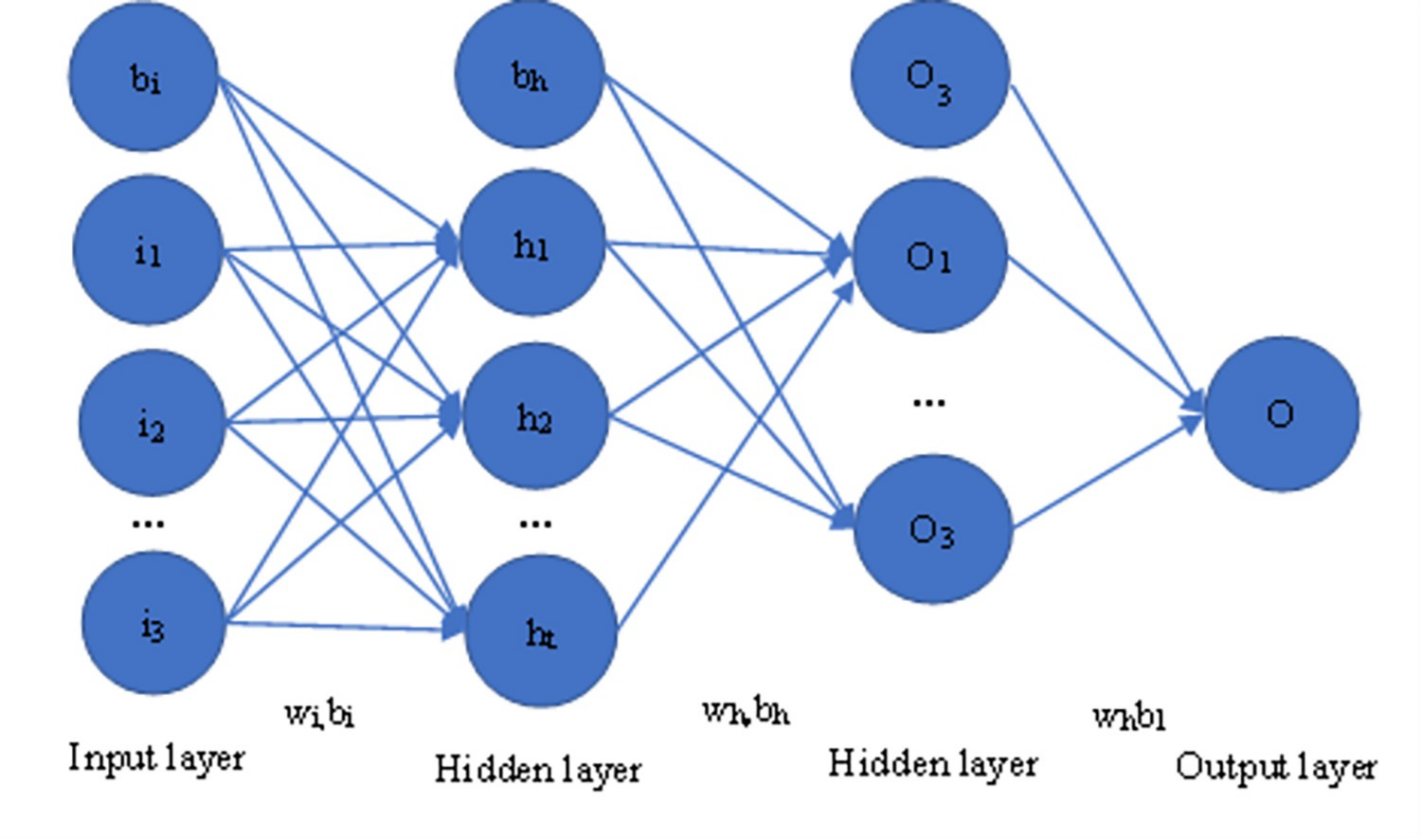
Structural model of the back-propagation neural network [30].

The learning process of the BPNN combines forward and backward propagation (Fig 1). With forward propagation, information moves from the input layer to the hidden layer where it is processed. Then, it is transmitted to the output layer. If the output is not what is expected, an error signal is sent backwards to the input layer so that the next input can learn from the previous cycle. Hence, the error is gradually reduced by adjusting the weights between the layers.

#### 3.1.1 Feed-forward neural network

Given *a*^(0)^ = *x*, the feed-forward propagation formula is as follows:

$$z^{\left(n\right)}=w^{\left(n\right)}a^{\left(n-1\right)}+b^{\left(n\right)},$$ (1)

$$a^{\left(n\right)}=f_{n}(z^{\left(n\right)}).$$ (2)

From this, we obtain

$$a^{\left(n\right)}=f_{n}(w^{\left(n\right)}a^{\left(n-1\right)}+b^{\left(n\right)}).$$ (3)

*n* is set as the number of neural-network layers, matching the number of neurons in the *N*th layer. *f*_*n*_(⋅) is the activation function of the *N*th layer, and *w*^(*n*)^ is the corresponding weight matrix. *b*^(*n*)^ as the bias value, *z*^(*n*)^ is the net input (net activity value) of the neurons in the *N*th layer, and *a*^(*n*)^ is the output (activity value) of the layer’s neuron. The entire network can be viewed as a composite function, *φ* = (*x*; *w*, *b*), and by taking vector *X* as the initial input, *a*^(0)^, information is transmitted layer-by-layer to obtain the active value of the entire function, *a*^(*N*)^, which includes the weights and biases of the network layer.

#### 3.1.2 Feedback neural network

Assuming that a given sample (*x*, *y*) is input into the neural network, stochastic gradient descent is used to learn the network parameters. Then, the network output is $\hat{y}$. After the feed-forward calculation of the forward propagation is complete, back-propagation is performed, wherein the error terms for each layer, δ^(n)^, are cycled back to the input:

$$For\;\forall n,\frac{\partial L(\cdot )}{\partial w^{\left(n\right)}}=\delta^{\left(n\right)}{\left(a^{\left(n-1\right)}\right)}^{T},$$ (4)

$$For\;\forall n,\frac{\partial L(\cdot )}{\partial b^{\left(n\right)}}=\delta^{\left(n\right)}.$$ (5)

Parameters are then updated as follows:

$$w^{\left(n\right)}⇐W^{\left(n\right)}-\alpha (\delta^{\left(n\right)}(a^{{\left(n-1\right)}^{T}})+\lambda w^{\left(n\right)}).$$ (6)

$$b^{\left(n\right)}⇐b^{\left(n\right)}-\alpha \delta^{\left(n\right)}.$$ (7)

When the error rate from the validation set no longer decreases, the trained weights, *w*, and biases, *b*, are the output. Above, *L*(⋅) is the loss function, *δ*^(*n*)^ is the error term of the *N*th layer, *α* is the learning rate, and *λ* is the regularization coefficient.

**3.1.3 BPNN forecasting time-series model.** In this study, a single-step prediction is used for our BPNN data processing structure:

y(t)=f(y(t−n),y(t−n+1),…y(t−1)). (8)

### 3.2 Core principles and computations of the LSTM

**Fig 2:**
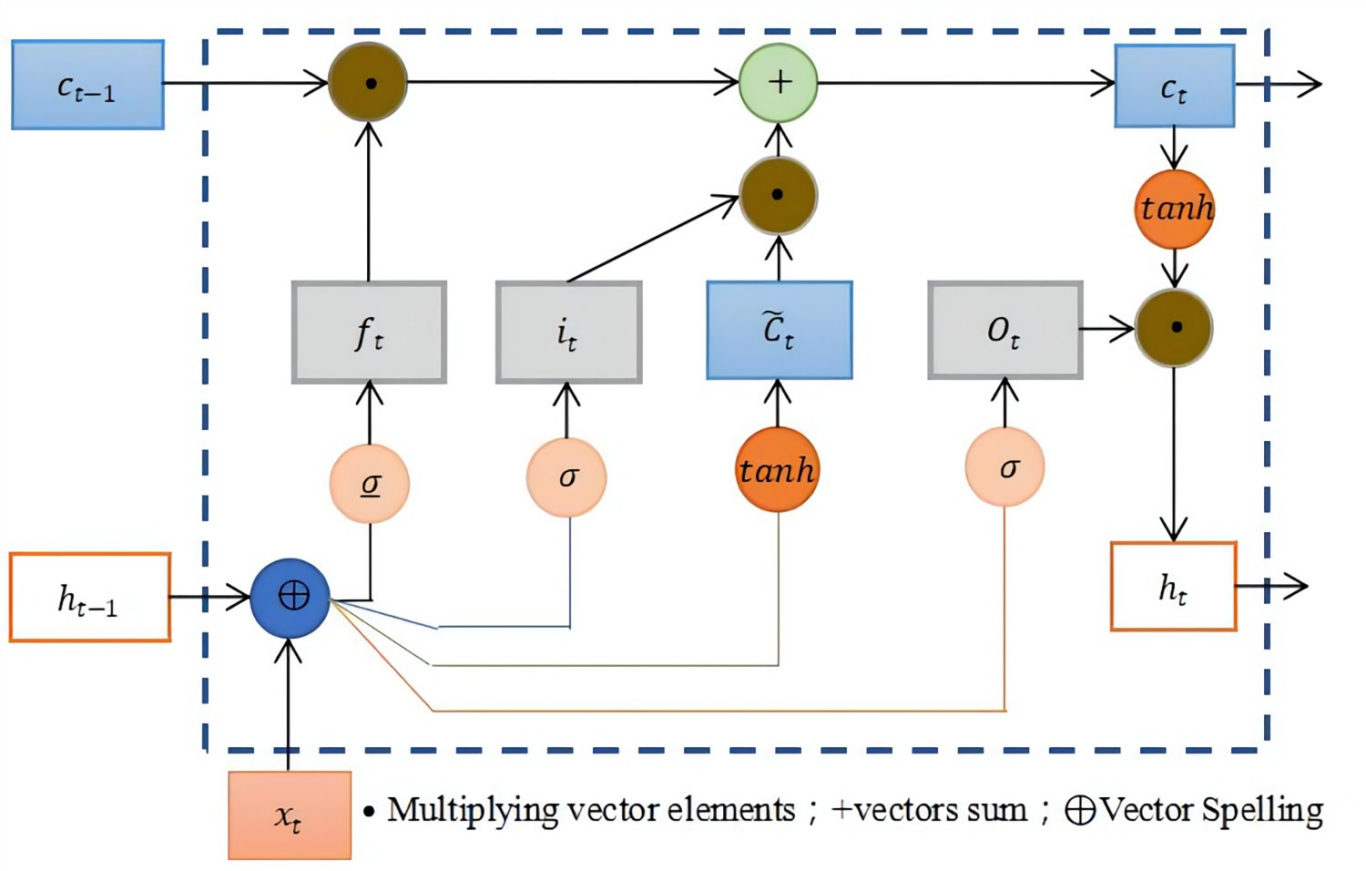
Structure of the long short-term memory neural network [30].

The input cycle and output mapping of an RNN at any given moment references all the data that have been processed until that moment, which results in a feedback network structure. When training an RNN, if the gradient disappears easily, then the learning rate is probably set too low, and training is likely to fail. The LSTM is a variant of the RNN, and the main differences include cell structure and operations (Fig 2). The LSTM solves the phenomenon of gradient disappearance or explosion by introducing a gating mechanism, and an LSTM cell consists of an input gate, an oblivion gate, and an output gate.

The LSTM network introduces a new internal state, *C*_*t*_∈*R*^*D*^, where *C*_*t*_ is the state of the cell at time *t*, which is dedicated to linear cyclic information transfer. Nonlinear outputting information is aligned with the external state of the hidden layer.

$$c_{t}=f_{t}\cdot c_{t-1}+i_{t}\cdot {\tilde{c}}_{t},$$ (9)

$$h_{t}=o_{t}\cdot \tanh\left(c_{t}\right),$$ (10)

$${\tilde{c}}_{t}=\tanh\left(W_{i}\cdot [h_{t-1},x_{t}]+b_{c}\right),$$ (11)

where *h*_*t*_ represents the external state of the hidden layer at time *t*, *tanh* is the activation function, and ${\tilde{c}}_{t}$ is the candidate state obtained nonlinearly. The oblivion gate, *f*_*t*_, determines the degree of influence of cell state *C*_*t*−1_ at moment (*t*−1) on cell state *c*_*t*_ at the current moment, *t*:

$$f_{c}=\sigma (w_{f}\cdot [h_{t-1},x_{t}]+b_{f}).$$ (12)

The weight matrix, *w*_*f*_, is multiplied by a matrix consisting of the previous and current moment values and a bias value, *b*_*f*_, which is processed by the activation function, σ, to obtain the result of the oblivion gate state calculation, *f*_*c*_.

The input gate, *i*_*t*_, determines the degree of influence of the current moment on state *C*_*t*_ of the network input cell, X_t_:

$$i_{t}=\sigma (w_{i}\cdot [h_{t-1},x_{t}]+b_{i}).$$ (13)

The input gate weight matrix, *w*_*i*_, is multiplied by a matrix consisting of the previous and current moment values and a bias value, *b*_*i*_, which is processed by the activation function, *σ*, to obtain the result of the input gate state calculation, *i*_*t*_.

The influence of the *O*_*t*_ control unit status, *c*_*t*_, on the current output value, *h*_*t*_, is calculated as follows:

$$O_{t}=\sigma (w_{0}\cdot [h_{t-1},x_{t}]+b_{0}).$$ (14)

At this point, the forward propagation process of the LSTM is complete.

### 3.3 BP-LSTM neural network

BPNNs have powerful nonlinear mapping capabilities, high self-learning and self-adaptability, good generalizability, and some fault tolerance. The three-layer version can approximate any nonlinear continuous function with arbitrary accuracy, which indicates that the BPNN is well-suited to solving problems with complex internal mechanisms. LSTMs are also suitable for time-series problems.

By combining the BPNN and LSTM, we apply a combined BP-LSTM to predict the prices of mangos. The main idea is like that of the gray neural network. In the BPNN, the input consists of a sample of variables, Xi, and labels, Y. The nonlinear relationship between the variables and labels is fitted and predicted by the BPNN. Hence, the time-series forecasting of multiple features is performed by the LSTM using the BPNN’s data.

### 3.4 Model evaluation indicators

RMSE, MAPE, and the R^2^ determination coefficient were used for the prediction accuracy evaluation [31]. The specific formulas are as follows:

$$RMSE=\sqrt{\frac{1}{m}\sum_{i=1}^{m}{\left({\hat{y}}_{i}-y_{i}\right)}^{2}},$$ (15)

$$MAPE=\frac{1}{n}\sum_{i=1}^{n}\left|\frac{{\hat{y}}_{i}-y_{i}}{y_{i}}\right|\times 100,$$ (16)

$$R^{2}=1-\frac{{\sum_{i}\left(\hat{y_{i}}-y_{i}\right)}^{2}}{{\sum_{i}\left(\bar{y_{i}}-y_{i}\right)}^{2}}.$$ (17)

Directional statistic (DS) is used for the directional forecasting accuracy of the competing models [32], defined as follows:

$$DS=\frac{1}{n}\sum_{i=1}^{n}F_{i}\times 100$$ (18)

Where *F*_*i*_ = 1, if $(y_{i+1}-y_{i})\times ({\hat{y}}_{i+1}-y_{t})\geq 0$ otherwise and represent the total number of predictions. Obviously, the lower the RMSE and MAPE values, the higher the prediction accuracy is. In contrast, the higher the R^2^ and DS values, the more accurate the direction prediction of the model. Next, we utilized the Diebold–Mariano (DM) statistic to compare the prediction errors of the two models [32].


$$DM=\frac{\bar{d}}{\sqrt{\mathrm{Var}\left(\bar{d}\right)}}$$ (19)

Where $\bar{d}=\frac{1}{n}\sum_{i=1}^{n}d_{i},d_{i}={\left(y_{i}-f_{1,i}\right)}^{2}-{\left(y_{i}-f_{2,i}\right)}^{2},\mathrm{Var}\left(\bar{d}\right)=\frac{1}{n}(\gamma_{0}+2\Sigma_{k=1}^{n}\gamma_{k})$ and $\gamma_{k}=\mathrm{Cov}\left(d_{i},d_{i-k}\right)$. *f*_1,*i*_ represents the predicted values obtained by the first model, whereas *f*_2,*i*_ obtained by the second model. The only assumption of DM statistic is that the two models have an equal number of predictions [32].

## 4 Empirical evidence and data analysis

This section reports on the accuracy and reliability measures of the proposed scheme as tested with a simulation. Performance comparisons were also made, and the results are provided.

### 4.1 Data processing

**Fig 3:**
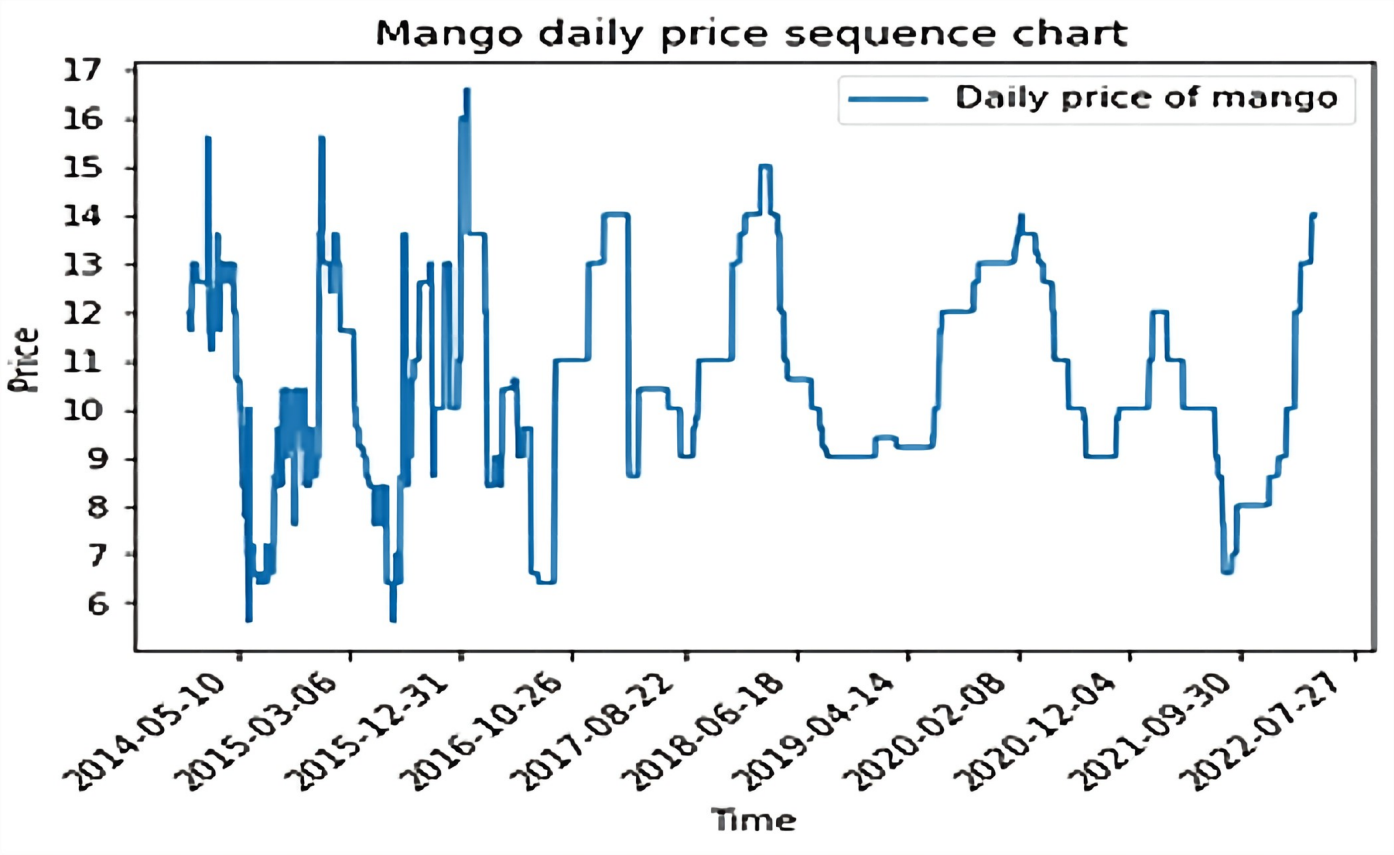
Daily price series of mangos from 2014-01-02 to 2022-04-18.

The raw daily mango price dataset was collected, but there were some missing values. Hence, linear interpolation was performed to fill the gaps. Afterwards, 3,029 total daily mango price data items were catalogued, and their price trends are shown in the Fig 3.

### 4.2 Model predictions and data analysis

#### 4.2.1 BPNN price forecasting

The BPNN structure consists of an input layer, an hidden layer, and an output layer. The input vector, X, is pre-processed to the hidden layer and then to the output layer to obtain the output vector, Y. The number of nodes in the hidden layer was set to 30; The training set consists of 80% of the data set, with the remaining 20% for testing. We tested the impact of various historical data on the prediction effect of the model in a comparative experiment to evaluate BPNN performance.

The smaller the value of RMSE and MAPE, the better, which means less error and more accurate. R^2^ stands for goodness of fit, and a larger value of R^2^ means a more suitable model. As shown in Table 1, Comprehensively compare the values of RMSE, MAPE and R^2^ of models with different time lags, the training results obtained by setting the partial time lag of the BPNN for two were optimal.

**Table 1:** Effects of different time lags on the prediction of back-propagation neural network models.

Time lag	1	2	3	4	5	6	7	8	9	10	11
RMSE	0.1954	**0.1712**	0.1811	0.1999	0.2109	0.2398	0.2014	0.2388	0.2251	0.2427	0.2518
MAPE	0.0090	0.0076	**0.0075**	0.0104	0.0122	0.0140	0.0080	0.0109	0.0102	0.0103	0.0122
R^2^	0.9854	**0.9888**	0.9875	0.9859	0.9830	0.9780	0.9845	0.9782	0.9807	0.9775	0.9758

*Note*: R^2^ = Determination coefficient; MAPE = Mean absolute percentage error RMSE = Root mean-square error.

**Fig 4:**
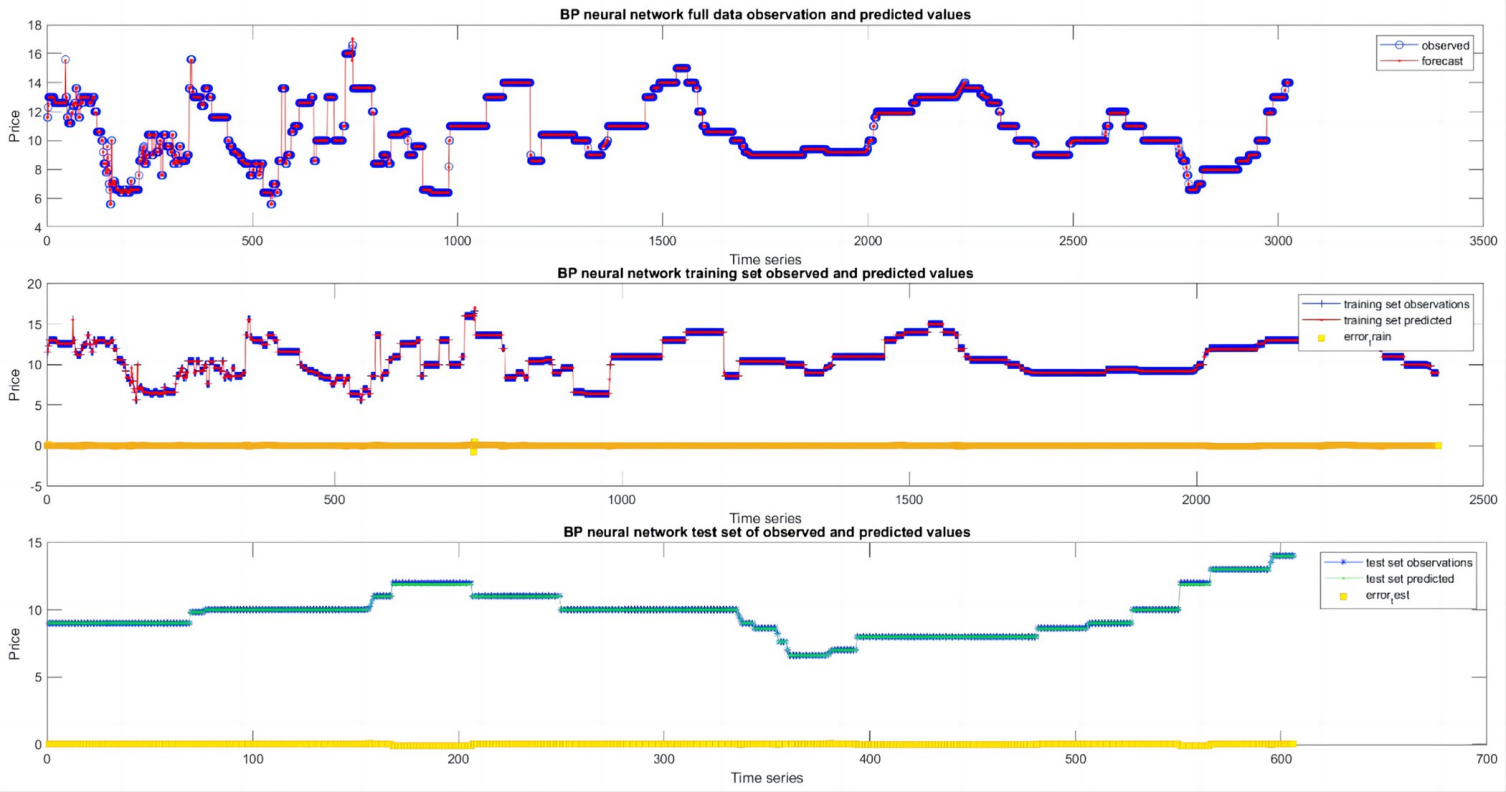
Combined observed and predicted values of the back-propagating neural network.

As shown in Fig 4, the BPNN was a good fit and was hence used to predict the basic trend of the daily mango prices, with only a small number of points resulting in large errors. Overall, the prediction results were reliable.

#### 4.2.2 LSTM price forecasting

**Fig 5:**
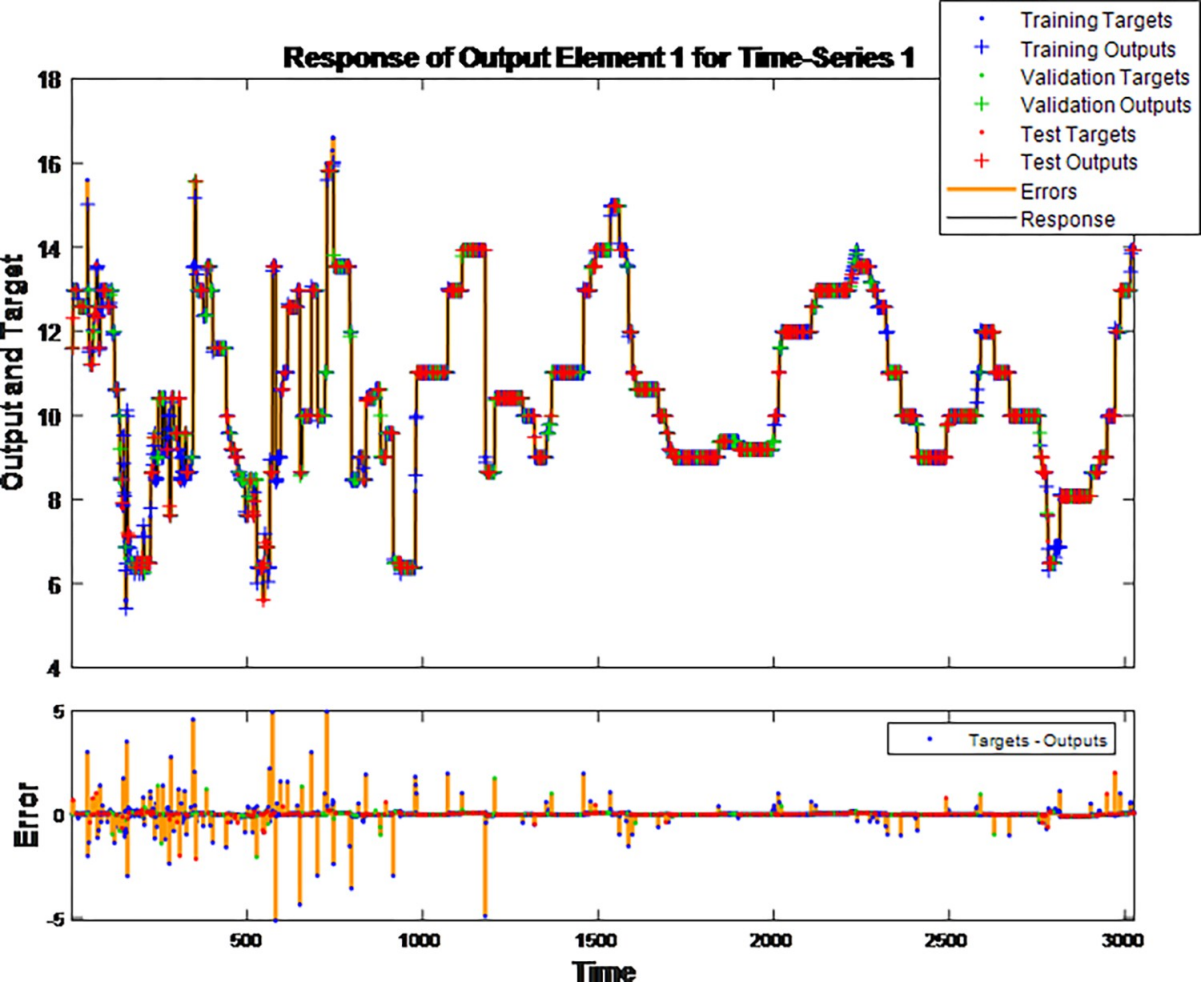
Long short-term memory training response plot.

The graph in Fig 5 shows the response plots obtained by the LSTM alone for time-series forecasting. Overall, the fit was good; however, the first half of the training data had a relatively large error, meaning that there was room for refinement.

#### 4.2.3 Combined BP-LSTM price forecasting

**Fig 6:**
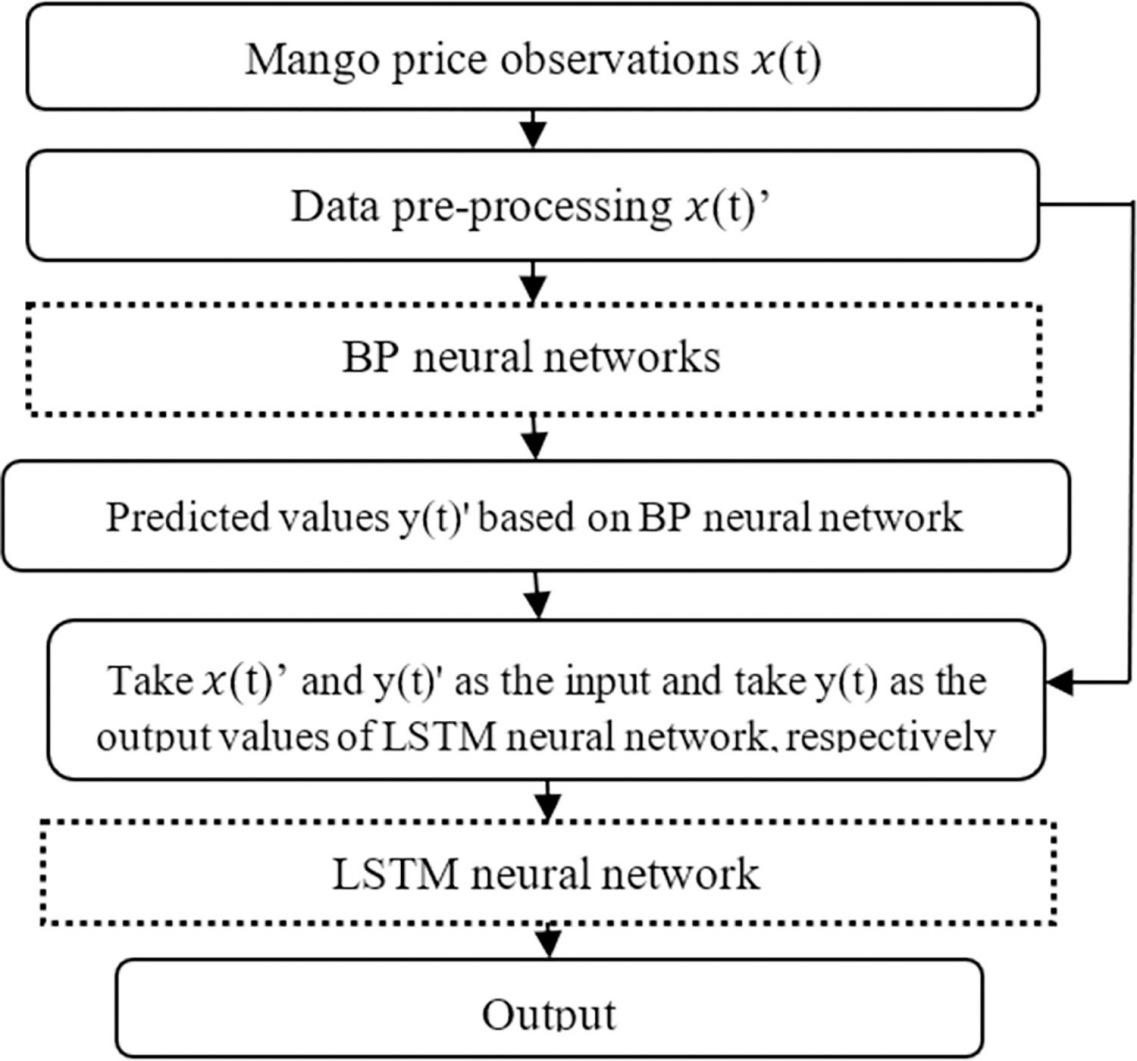
Structure of the combined back-propagation long short-term memory model.

**Fig 7:**
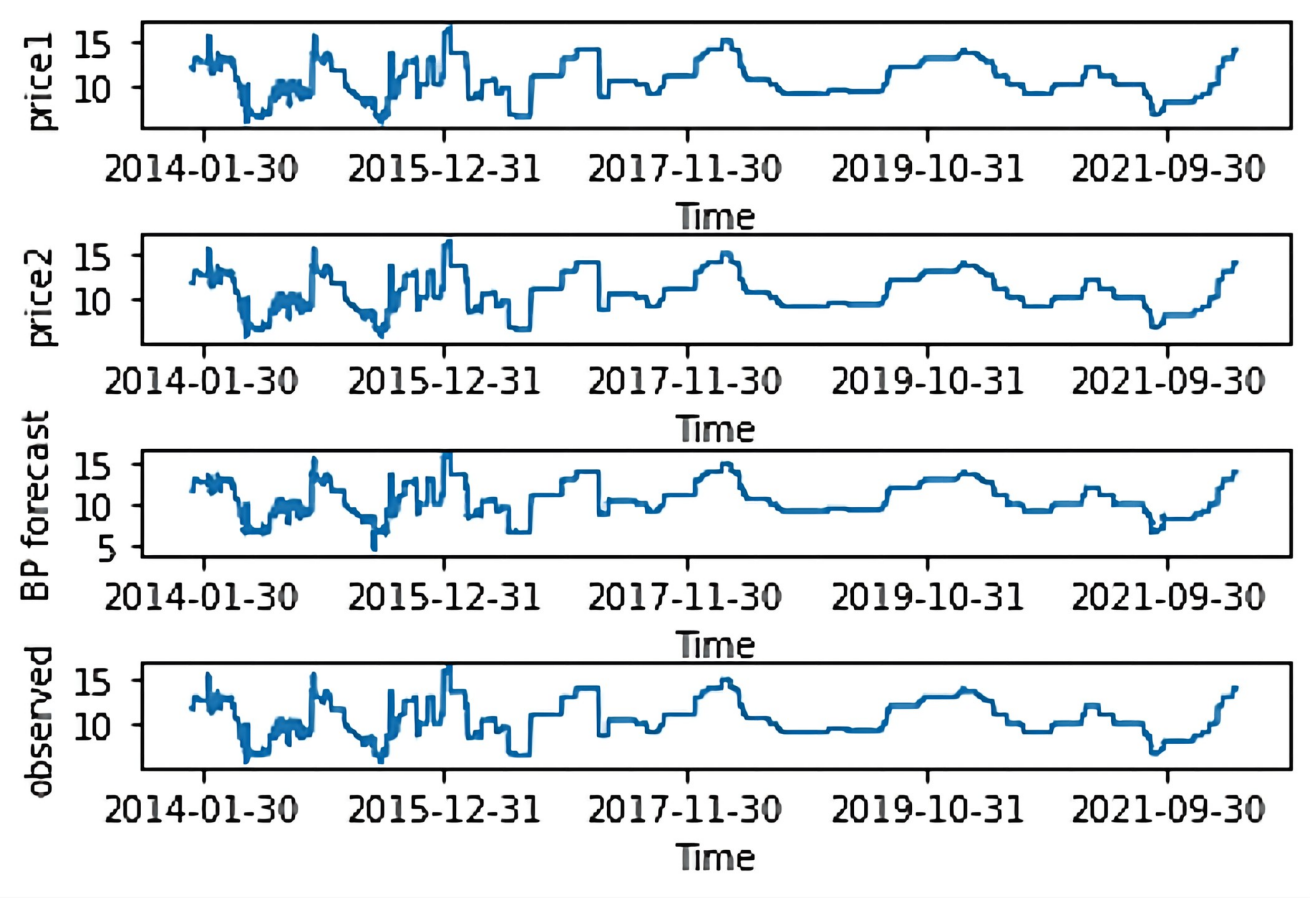
Data used in the back-propagation long short-term memory model.

The structure of the BP-LSTM model is shown in Fig 6. Various input nodes were selected according to their effects on BP model accuracy. As shown in Fig 7, two input nodes were found to be optimal. Historical Prices 1 and 2 were then input, and the BPNN performed single-step time series predictions. Then, based on the original input data, the BPNN’s predicted values were fed to the LSTM for multiple-input price prediction.

Like the BPNN, the LSTM used 80% of the input data for training and 20% for the testing set. The network layers were generated using Matlab software, and the parameters were fine-tuned and optimized.

**Fig 8:**
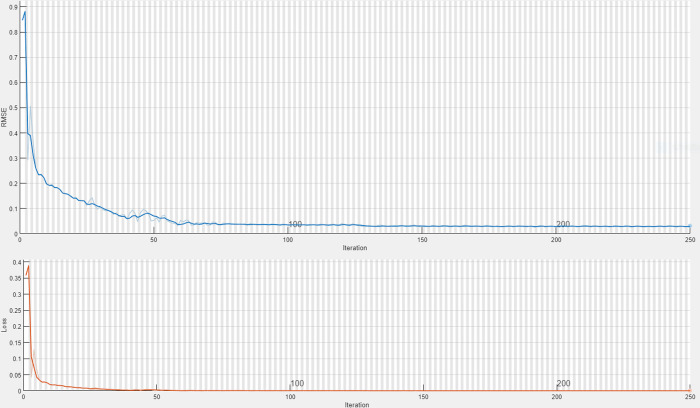
BP-LSTM model training process.

Fig 8 provides a visualization of the BP-LSTM training process. Note that overfitting was suppressed by data normalization, operational standardization, and dropout layer application. There is no significant increase in the value of the loss function over time, indicating that the training process is good. BP-LSTM’s RMSE was below 0.1 halfway through the iterative process, which is clearly better than the accuracy of the BPNN alone.

**Fig 9:**
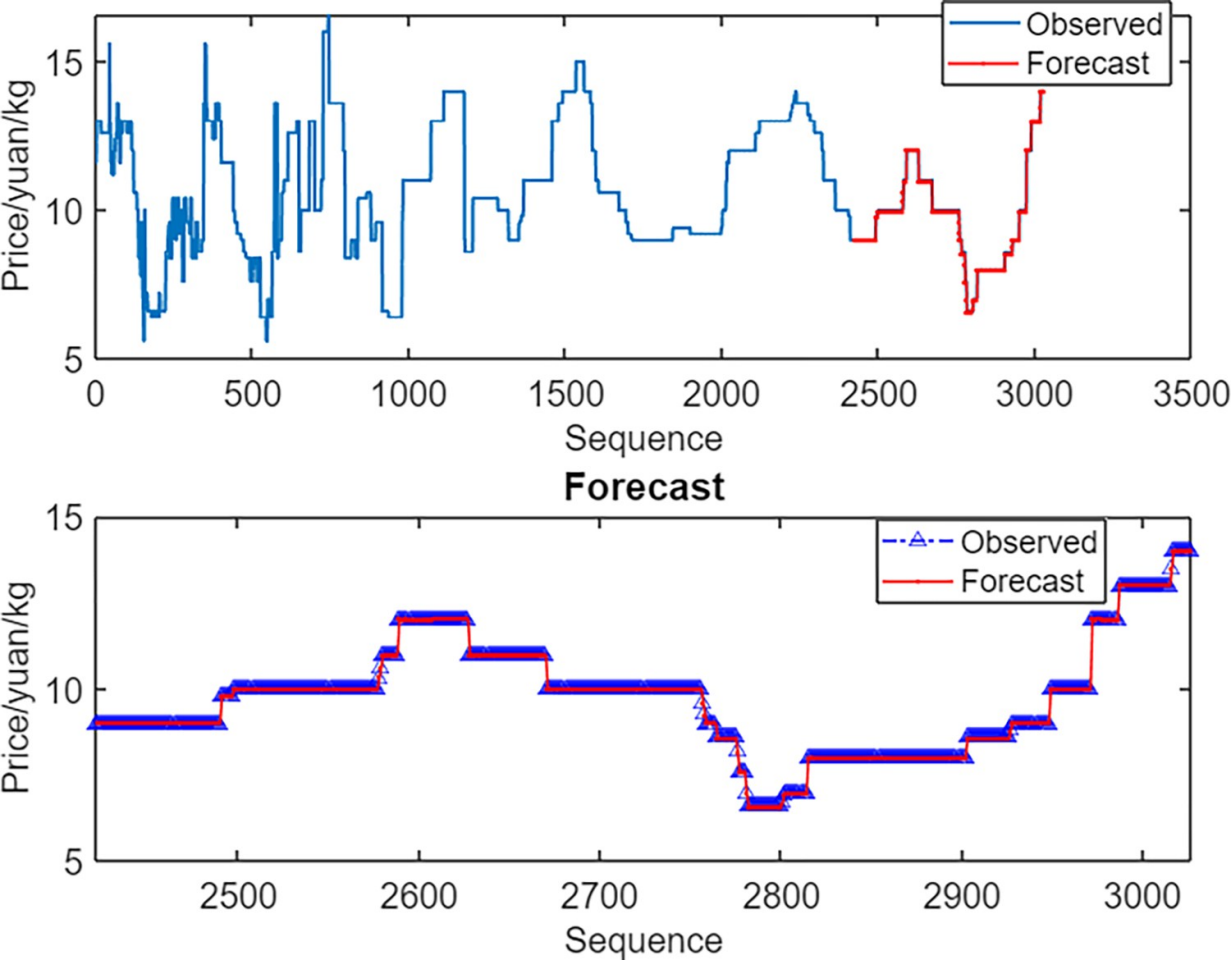
BP-LSTM model prediction results.

**Fig 10:**
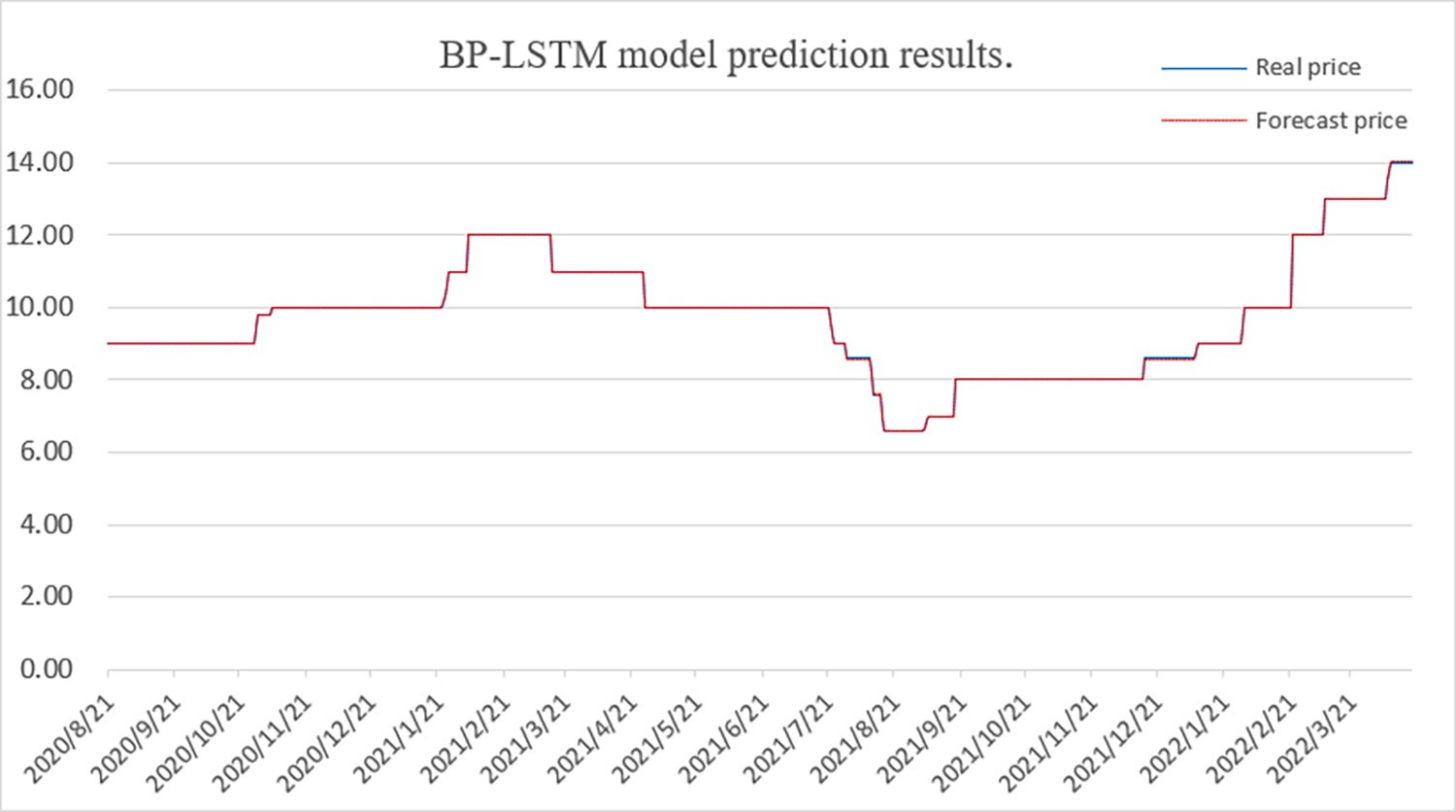
BP-LSTM model test set prediction results.

Fig 9 shows the BP-LSTM model prediction results, indicating that the observed and predicted prices are very close. Fig 10 displays the BP-LSTM model test set prediction results, further demonstrating the accuracy of the model.

Table 2 shows a comparison of the values used to test the model criteria.

**Table 2:** Comparison of parameters of different model prediction levels.

Model	RMSE	MAPE	R^2^
BP	0.1707	0.0095	0.9888
LSTM	0.3525	0.0101	0.9717
BP-LSTM	0.0175	0.0014	0.9998

*Note*: R^2^ = Determination coefficient; MAPE = Mean absolute percentage error; RMSE = Root mean-square error.

As shown in Table 2, the combined BP-LSTM model achieved values of 0.0175 and 0.0014 for RMSE and MAPE, respectively, both of which are lower than those for the BPNN and LSTM alone. The BP-LSTM’s R^2^ value was 0.9998 higher than the values of 0.9888 and 0.9717 predicted by the BPNN and LSTM separately, indicating that the generalizability of the combined BP-LSTM model is superior. Much of the improvement is traceable to the BPNN’s network prediction stage. Notably, the amount of input data also affects the prediction results as too few input nodes will extract less information. However, too many nodes allow too much irregular information. Based on reasonable BPNN predictions, the LSTM can extract more features, which further improves the prediction accuracy.

From Table 2, we can see that BP prediction model and BP-LSTM model are superior to LSTM model in the prediction level indicators RMSE, MAPE and goodness of fit parameter R^2^. Based on this, we further compare the performance of BP model and BP-LSTM model in terms of directional predictions. The directional predictions are more suitable for business purpose because the investors are more interested in the trend of the market.

As shown in Table 3, the DS test value of BP-LSTM model is greater than that of BP prediction model, indicating that BP-LSTM prediction model performs better than BP model in directional prediction.

**Table 3:** Comparison of directivity prediction accuracy of different models.

Model	DS
BP	96.86
BP-LSTM	100

As shown in Table 4, under different error measurement methods, the p-value of the model DM test is less than 0.05, indicating that the model prediction effect is significantly different. The DM value is greater than 0, indicating that the BP-LSTM model prediction result is significantly better than the BP model according to the test rules.

**Table 4:** Comparison of Diebold-Mariano test values of models under different error measurement methods.

MAD	Tested Model	BP	BP-LSTM	MAE	Tested Model	BP	BP-LSTM
	BP		3.450 (0.001)		BP		12.831 (0.000)
	BP-LSTM				BP-LSTM		

*Note*: MAD = Median absolute deviation; MAE = Mean absolute error.

In conclusion, the BP-LSTM prediction model proposed in this study performs better than a single model.

## 5 Discussion

Price forecasting is a very meaningful thing that can help us make better behavioral decisions. From the economic point of view, price forecast can provide a basis for price decision and economic decision. From the perspective of agricultural product development, it is beneficial to make reasonable arrangement and adjustment of production to measure and judge the trend of price change. From the logistics perspective, adjust the logistics plan and reasonably arrange the logistics activities.

In the process of the reference study in the early stage of this study, we found that the predicted results of the price prediction model based on deep learning are generally better than the traditional price data prediction methods. With the deepening of research, a large number of combined forecasting models based on traditional forecasting methods and deep learning single forecasting model have emerged. These combined models can be roughly divided into two categories, one is the combination of traditional prediction methods and deep learning prediction models, and the other is the combination of different kinds of deep learning single prediction models. The modes of model combination include sequential combination, nesting and so on. Because the combined model combines the advantages and performance of multiple models, the combined model is generally superior to the single model in terms of forecasting performance. Based on this, we conducted a research on price prediction of characteristic agricultural products. We selected mangos with regional representation in China as the research object and proposed a new price prediction model for their price prediction, aiming at more accurate mango price prediction. To sum up, the contributions of this paper mainly include the following aspects.

1. In terms of research objects, the research object of this paper is mango price prediction. Mango is a regional characteristic fruit, which plays an important role in promoting regional characteristic products to develop regional characteristic economy. Therefore, this study has certain reference value for the research of developing regional characteristic economy from the economic perspective.
2. This study proposes an innovative portfolio model for price forecasting. The BP-LSTM model proposed in this paper is superior to the single models BP and LSTM that constitute the model in terms of goodness of fit and prediction accuracy.
3. From the perspective of data, the experimental data sources used in this paper are official and orthodox, with strong representativeness and persuasiveness; Large amount of data, relatively consistent data and strong reliability. In terms of model performance comparison, this paper provides the experimental data shown in Tables 2–4, and the experimental results are intuitive and accurate.

## 6 Conclusions

With the development of the supply chain of agricultural products, the forecast of agricultural product’s price provides a scientific basis for the decision of reducing cost and increasing efficiency of agricultural products supply chain. Especially for the regional characteristic fruits used to support the development of the region, fruit price prediction is of great significance to guide the development of the fruit industry. However, there are few relevant studies at present, and the accuracy of traditional price prediction is poor. Under these conditions, researchers consider mango as the research carrier, combined with neural network prediction model to carry out research. In this study, a combined prediction model based on BP neural network and LSTM neural network is proposed, which takes the characteristic fruit industry as the foothold and the mango as the representative of the characteristic fruit, and predicts the price of mango. Using the daily price data of mangoes in 8 years and 4 months of a large fruit wholesale trading center in China as an example, the prediction performance of the proposed BP-LSTM combined prediction model is verified. The research results show that:

- The combined BP-LSTM prediction model constructed by combining the characteristics of BP neural network model and LSTM neural network model is more accurate under the test of the model accuracy evaluation index system established with the parameters of RMSE, MAPE and R^2^.
- Based on the daily price data collected for mangos, the average domestic price of mangoes in China is 10.56 RMB/kg, and the daily fluctuation falls within 5.6–16.6 RMB/kg. Notably, in China, the natural mango ripening season begins in July, at which time they are the least costly to cultivate, and they are in greater supply. Hence, according to the law of market prices, the daily price trend moves downward in July and lasts for a period of one or two months, followed by an upward trend in the nonripe season.
- Our BP-LSTM price prediction provided more accurate, one-step price predictions, and the results are judged to be useful in providing a basis for making short-term price decisions to reduce risks.
- Notably, the empirical data used in this paper originated from China, and their utility for making accurate predictions using the proposed BP-LSTM model is currently not generalizable outside of that region. Information from India should be examined next, using this tool BP-LSTM to check its wider application. Moreover, it has been noted that additional neural-network combinations may work better in different regions, which certainly makes efforts like this worthwhile in terms of enabling flexible decision-making with creative and representative modeling tools.

## Supporting information

S1 Data (ZIP)
