# Supplementary material for: Characteristic mango price forecasting using combined deep-learning optimization model
Source: PLoS One. 2023 Apr 13;18(4):e0283584. doi: 10.1371/journal.pone.0283584 (PMC10101496; doi:10.1371/journal.pone.0283584)
Supplement: S1 Data — (ZIP) [file pone.0283584.s001.zip › Experimental data and related Codes (English Version)/DS and DM test and data adjustment supplement/Directional Statistics (DS).html]

Directional Statistics (DS)


In [16]:

```
data=read.csv("C:\\Users\\y'chao\\Desktop\\dsanddm.csv")
```

In [17]:

```
n=length(data$actual)
```

In [34]:

```
Ft.BP=sum(((data$actual[2:n]-data$actual[1:n-1])*(data$pred.BP[2:n]-data$actual[1:n-1])>=0)*1)/(n-1)*100
Ft.BP
```

96.8595041322314

In [33]:

```
Ft.LSTM=sum(((data$actual[2:n]-data$actual[1:n-1])*(data$pred.LSTM[2:n]-data$actual[1:n-1])>=0)*1)/(n-1)*100
Ft.LSTM
```

98.1818181818182

In [32]:

```
Ft.BpLstm=sum(((data$actual[2:n]-data$actual[1:n-1])*(data$pred.BpLstm[2:n]-data$actual[1:n-1])>=0)*1)/(n-1)*100
Ft.BpLstm
```

100
