# Supplementary material for: Characteristic mango price forecasting using combined deep-learning optimization model
Source: PLoS One. 2023 Apr 13;18(4):e0283584. doi: 10.1371/journal.pone.0283584 (PMC10101496; doi:10.1371/journal.pone.0283584)
Supplement: S1 Data — (ZIP) [file pone.0283584.s001.zip › Experimental data and related Codes (English Version)/DS and DM test and data adjustment supplement/DM_Test.html]

DM\_Test


In [78]:

```
from scipy.stats import t
import collections
import pandas as pd
import numpy as np

def dm_test(actual, pred1, pred2, method="MSE",v = 1):
    error1 = []
    error2 = []
    d  = []

    actual = list(pd.Series(actual).apply(lambda x: float(x)))
    pred1 = list(pd.Series(pred1).apply(lambda x: float(x)))
    pred2 = list(pd.Series(pred2).apply(lambda x: float(x)))
    
    n = float(len(actual))
    if (method == "MSE"):
        for actual,pred1,pred2 in zip(actual,pred1,pred2):
            error1.append(abs(actual - pred1))
            error2.append(abs(actual - pred2))
        for error1, error2 in zip(error1, error2):
            d.append(error1 - error2)
   
    elif (method == "MAD"):
        for actual,pred1,pred2 in zip(actual,pred1,pred2):
            error1.append((actual - pred1)**2)
            error2.append((actual - pred2)**2)
        for error1, error2 in zip(error1, error2):
            d.append(error1 - error2)
    elif (method == "MAPE"):
        for actual,pred1,pred2 in zip(actual,pred1,pred2):
            error1.append(abs((actual - pred1)/actual))
            error2.append(abs((actual - pred2)/actual))
        for error1, error2 in zip(error1, error2):
            d.append(error1 - error2)
   
    md = pd.Series(d).mean()
    def autocovariance(a1, N, b, a2):
        ac = 0
        n = float(N)
        for i in np.arange(0, N-b):
              ac += ((a1[i+b])-a2)*(a1[i]-a2)
        return (1/(n))*ac
    gamma = []
    for lag in range(0,v):
        gamma.append(autocovariance(d,len(d),lag,md)) # 0, 1, 2
    vd = (gamma[0] + 2*sum(gamma[1:]))/n
    DM=md*vd**(-0.5)
    har=((n+1-2*v+v*(v-1)/n)/n)**(0.5)
    DM = har*DM
    p_value = 2*t.cdf(-abs(DM), df = n - 1)
    dr = collections.namedtuple('dr', 'DM p_value')
    DMtest= dr(DM = DM, p_value = p_value)
    
    return DMtest
```

In [79]:

```
import pandas as pd

data=pd.read_excel("C:\\Users\\y'chao\\Desktop\\dsanddm.xlsx")
```

In [80]:

```
actual = list(data.values[:,0])
pred1= list(data.values[:,1])
pred2 = list(data.values[:,2])
pred3 = list(data.values[:,3])

DMtest = dm_test(actual,pred1,pred2, method="MAD")
print(DMtest)
DMtest = dm_test(actual,pred1,pred3, method="MAD")
print(DMtest)
DMtest = dm_test(actual,pred2,pred3, method="MAD")
print(DMtest)

DMtest = dm_test(actual,pred1,pred2, method="MSE")
print(DMtest)
DMtest = dm_test(actual,pred1,pred3, method="MSE")
print(DMtest)
DMtest = dm_test(actual,pred2,pred3, method="MSE")
print(DMtest)

DMtest = dm_test(actual,pred1,pred2, method="MAPE")
print(DMtest)
DMtest = dm_test(actual,pred1,pred3, method="MAPE")
print(DMtest)
DMtest = dm_test(actual,pred2,pred3, method="MAPE")
print(DMtest)
```

```
dr(DM=5.291181938580626, p_value=1.7014932055876476e-07)
dr(DM=3.45003479869866, p_value=0.0005995271733038646)
dr(DM=2.93073800496079, p_value=0.003509195609026224)
dr(DM=24.99870842971177, p_value=2.8107273086562384e-95)
dr(DM=12.831346048318743, p_value=1.6655970297837505e-33)
dr(DM=7.1256681081586875, p_value=2.9574588006078153e-12)
dr(DM=20.630326743475642, p_value=5.27015722432415e-72)
dr(DM=13.66049732586699, p_value=3.1904965029313843e-37)
dr(DM=7.817968179496306, p_value=2.3933397560908505e-14)
```
